# Blood–spinal cord barrier breakdown and pericyte reductions in amyotrophic lateral sclerosis

**DOI:** 10.1007/s00401-012-1039-8

**Published:** 2012-09-01

**Authors:** Ethan A. Winkler, Jesse D. Sengillo, John S. Sullivan, Jenny S. Henkel, Stanley H. Appel, Berislav V. Zlokovic

**Affiliations:** 1 Center for Neurodegeneration and Regeneration, Zilkha Neurogenetic Institute, University of Southern California, Room: 101, 1501 San Pablo Street, Los Angeles, CA 90089 USA; 2 Department of Neurology, Methodist Neurological Institute, The Methodist Hospital Research Institute, The Methodist Hospital, Houston, TX USA

**Keywords:** Amyotrophic lateral sclerosis, Pericytes, Vascular injury, Blood–brain barrier, Blood–spinal cord barrier

## Abstract

The blood–brain barrier and blood–spinal cord barrier (BSCB) limit the entry of plasma components and erythrocytes into the central nervous system (CNS). Pericytes play a key role in maintaining blood–CNS barriers. The BSCB is damaged in patients with amyotrophic lateral sclerosis (ALS). Moreover, transgenic ALS rodents and pericyte-deficient mice develop BSCB disruption with erythrocyte extravasation preceding motor neuron dysfunction. Here, we studied whether BSCB disruption with erythrocyte extravasation and pericyte loss are present in human ALS. We show that 11 of 11 cervical cords from ALS patients, but 0 of 5 non-neurodegenerative disorders controls, possess perivascular deposits of erythrocyte-derived hemoglobin and hemosiderin typically 10–50 μm in diameter suggestive of erythrocyte extravasation. Immunostaining for CD235a, a specific marker for erythrocytes, confirmed sporadic erythrocyte extravasation in ALS, but not controls. Quantitative analysis revealed a 3.1-fold increase in perivascular hemoglobin deposits in ALS compared to controls showing hemoglobin confined within the vascular lumen, which correlated with 2.5-fold increase in hemosiderin deposits (*r* = 0.82, *p* < 0.01). Spinal cord parenchymal accumulation of plasma-derived immunoglobulin G, fibrin and thrombin was demonstrated in ALS, but not controls. Immunostaining for platelet-derived growth factor receptor-β, a specific marker for CNS pericytes, indicated a 54 % (*p* < 0.01) reduction in pericyte number in ALS patients compared to controls. Pericyte reduction correlated negatively with the magnitude of BSCB damage as determined by hemoglobin abundance (*r* = −0.75, *p* < 0.01). Thus, the BSCB disruption with erythrocyte extravasation and pericyte reductions is present in ALS. Whether similar findings occur in motor cortex and affected brainstem motor nuclei remain to be seen.

## Introduction

Amyotrophic lateral sclerosis (ALS) is the most common motor neuron disorder with an incidence of approximately 2–3 cases annually per 100,000 people [18, 37, 43, 69]. Approximately 90 % of cases are sporadic and of unknown molecular etiology. The remaining 10 % of cases are inherited as autosomal dominant mutations in as many as 12 genes, including those encoding for superoxide dismutase 1 (*SOD1*), TAR DNA binding protein (*TDP*-*43*), fusion in sarcoma (*FUS*), angiogenin (*ANG*) and optineurin (*OPTN*) [1, 22], and an expanded hexanucleotide repeat of GGGGCC in a noncoding region of the C9Orf72 gene [15, 55]. Motor neuron injury and loss appear to result from the interaction of pathological processes arising from within neurons and neighboring non-neuronal cells. Non-cell autonomous sources of motor neuron injury have been identified in transgenic ALS mouse models [5, 9, 12, 34, 65] and human neuronal co-cultures [30], and have largely focused on glial cell populations [52].

In contrast to leaky systemic capillaries [44], the blood–brain barrier (BBB) and blood–spinal cord barrier (BSCB) prevent the entry of toxic circulating molecules, erythrocytes and leukocytes into the central nervous system (CNS) [62, 70, 71]. The BBB/BSCB is damaged in ALS subjects as shown through analysis of albumin and other serum-derived proteins in the cerebrospinal fluid (CSF) and measurements of albumin CSF/serum quotients [2, 3, 10, 29, 40, 45, 53, 57], as well as postmortem pathologic analyses of spinal cord or motor cortex tissue [17, 20, 21, 31, 32, 58]. Consistent with findings in humans, transgenic rodents expressing mutant isoforms of human SOD1 develop a spontaneous BSCB breakdown [24–26, 46, 48, 66, 67]. In *SOD1* transgenic mice, BSCB breakdown causing extravasation of erythrocytes precedes motor symptoms and neuronal loss [46, 66, 67]. Recent studies in transgenic rodents with dysfunctional signaling in pericytes have demonstrated that pericytes play a key role in maintaining the integrity of the BBB and BSCB [4, 6, 7, 14, 63]. For example, mice with deficient platelet-derived growth factor receptor-β (*Pdgfrβ*) signaling in pericytes resulting in pericyte loss develop chronic BBB and/or BSCB disruption with accumulation of plasma proteins and erythrocyte extravasation preceding neuronal injury [6, 63].

In the present study, we utilized high resolution histopathologic analyses to determine whether BSCB disruption in humans with ALS results in extravasation of erythrocytes in motor neuron dense regions of the spinal cord, and whether BSCB disruption in human ALS is associated with a reduction in the pericyte population. We show not only that capillary leakage of erythrocytes and plasma proteins is present in ALS, but also that vascular rupture coincides with reductions in capillary pericyte populations in human ALS spinal cord tissue specimens. These findings raise a number of questions regarding the significance and/or contributory role of vascular dysfunction in ALS pathogenesis.

## Materials and methods

### Human subjects

Written consent was obtained and approved by The Methodist Hospital from all human subjects utilized in this study prior to death. The postmortem interval ranged between 5 and 16 h for all tissue samples. Postmortem cervical spinal cord tissue samples were obtained from subjects with a definite diagnosis of ALS according to the WFN El Escorial/Airlie criteria. Following cervical spinal cord isolation, tissue samples were immediately snap frozen and stored at −80 °C, unless otherwise indicated. In total, tissue samples from eight subjects with sporadic sALS, three subjects with familial (fALS) and five non-neurodegenerative disease controls (NNDC) were utilized for all studies (see Table 1 for details). The cause of death in all ALS patients was respiratory failure, whereas the cause of death in NNDC cases was either cardiac failure or respiratory failure.

**Table 1:** Demographic and clinical features of human subjects

	NNDC (*n* = 5)	sALS (*n* = 8)	fALS (*n* = 3)
Gender (M/F)	3/2	5/3	2/1
Age of diagnosis (mean ± SD)	–	56.5 ± 13.3	56.4 ± 3.0
Disease duration (mean ± SD)	–	4.6 ± 4.7	3.1 ± 2.4
Age of death (mean ± SD)	61.2 ± 10.0	60.7 ± 12.5	59.5 ± 1.8

### Perivascular hemosiderin deposits

To analyze perivascular Prussian blue-positive hemosiderin deposits, tissues were cryosectioned at a thickness of 14 μm, fixed with immersion in ice cold acetone and stored at −20 °C. Sections were then rehydrated in PBS and blocked in 10 % normal swine serum. Sections were then incubated in goat anti-human podocalyxin (1:100, R&D systems, Minneapolis, MN, USA). Sections were then incubated with horseradish peroxidase (HRP)-conjugated rabbit anti-goat secondary antibody (Bio-Rad, Hercules, CA, USA). Podocalyxin positive vessels were subsequently visualized utilizing 3-3′-diaminobenzidine (DAB) peroxidase substrate kit (Vector Laboratories, Burlingame, CA, USA). Prussian blue staining was then performed as we previously described [6, 66, 67]. Briefly, sections were incubated in 5 % potassium ferrocyanide and 5 % hydrochloric acid solution (diluted 1:1) for 30 min at 37 °C. Sections were then rinsed in water, mounted in Cytoseal XYL mounting media (Thermo Scientific, Waltham, MA, USA) and coverslipped.

To quantify perivascular Prussian-blue positive hemosiderin deposits, a minimum of five 1.27 × 0.95 mm images confined to the anterior horn gray matter were randomly taken using a Leica DMI6000 inverted epifluorescent microscope (Leica Instruments, Nussloch, Germany) per section. Six non-adjacent tissue sections (~100 μm apart) were analyzed per subject. Perivascular Prussian-blue positive hemosiderin deposits were manually counted and expressed per mm^2^ spinal cord.

### Hematoxylin and eosin staining

Following spinal cord isolation, tissue samples were fixed in formalin and embedded in paraffin. Embedded tissue was sectioned using a Leica RM2125 microtome at a thickness of 5 μm. Sections were deparaffinized with xylene and rehydrated to distilled water with serial ethanol washes. Hematoxylin and eosin staining was then performed as described by the manufacturer (FD NeuroTechnologies, Columbia, MD). Sections were subsequently coverslipped using Cytoseal XYL mounting media (Thermo Scientific) and imaged using a Leica DMI6000 inverted epifluorescent microscope (Leica Instruments).

### Immunofluorescent analysis

Snap frozen cervical spinal cords were embedded in optimal cutting temperature (OCT) compound (Tissue-Tek, Torrance, CA, USA). Embedded tissues were cryosectioned at a thickness of 14 μm, fixed with immersion in ice cold acetone and stored at −20 °C. Sections were rehydrated in phosphate buffered saline (PBS) and blocked in 10 % normal swine serum (Vector Laboratories, Burlingame, CA, USA) for 1 h at room temperature. Sections were then incubated overnight at 4 °C in the following primary antibodies: goat anti-platelet derived growth factor β (PDGFRβ) (10 μg/mL, R&D systems), goat anti-hemoglobin (10 μg/ml, R&D systems), mouse anti-glycophorin A (CD235a) (1:100, Dako, Carpinteria, CA, USA), rabbit anti-fibrinogen (1:100, Dako), goat anti-thrombin (1:50, Santa Cruz Biotechnology, Santa Cruz, CA, USA) and mouse anti-neuronal specific antigen A60 (NeuN) (EMD Millipore, Billerica, MA, USA). To visualize pericytes, sections were incubated in bovine anti-goat Cy3 (Jackson ImmunoResearch Laboratories, Inc., West Grove, PA, USA) diluted 1:100 to detect PDGFRβ. To detect erythrocyte-derived products, sections were incubated in bovine anti-goat Cy3 (Jackson Laboratories) and donkey-anti rabbit Alexa Fluor 488 (Jackson Laboratories) to detect hemoglobin and CD235a, respectively. To detect plasma proteins, sections were incubated in bovine anti-goat Cy3 (Jackson Laboratories) and donkey-anti rabbit Alexa Fluor 488 (Jackson Laboratories) to detect thrombin and fibrin, respectively. To visualize neurons, sections were incubated in donkey anti-mouse Alexa Fluor 488 (Jackson Laboratories) to visualize NeuN-positive cells. To visualize endothelial cells, sections were incubated in fluorescein labeled Ulex Europaeus Agglutinin I lectin (UEA-1) (Vector Laboratories) or biotinylated labeled UEA-1 (Vector Laboratories) followed by incubation in DyLight 649 conjugated streptavidin (Vector Laboratories). Following immunodetection, sections were incubated in 1 % Sudan Black B (Sigma Aldrich, St. Louis, MO, USA) diluted in 70 % ethanol for 10 min at room temperature.

Tissue sections were mounted with fluorescent mounting media (Dako) and coverslipped. All slides were scanned with a custom built Zeiss 510 meta confocal laser scanning microscope with a Zeiss Apochromat 25×/0.8 NA water immersion objective (Car Zeiss Microimaging Inc., Thornwood, NY, USA). A 488 nm argon laser was used to excite Alexa Fluor 488 and fluorescein and the emission was collected through a 500–550 nm band pass (bp) filter. A 543 nm HeNe laser was used to excite Cy3 and the emission was collected through a 560–615 nm bp filter. A 633 nm HeNe laser was used to excite DyLight 649 and the emission was collected through a 650–700 nm bp filter.

### Image analysis

All image analyses were conducted utilizing NIH ImageJ software. For all analyses, a field size of 420 × 420 μm was utilized and 10–12 μm maximum projection z-stack images were reconstructed. For all studies, five randomly selected fields confined within the anterior horn gray matter per section from six non-adjacent sections (~100 μm apart) were analyzed from each tissue specimen. For pericyte coverage analysis, PDGFRβ-positive surface area was determined by the ImageJ Area measurement tool and divided by the lectin-positive capillary surface area as we previously described [6, 61, 63]. Quantification of extravascular hemoglobin deposits were performed as we previously described for plasma proteins with several modifications [6, 66, 67]. Intravascular hemoglobin-positive immunofluorescent signal (hemoglobin-positive staining which co-localized with lectin positive capillaries) was subtracted from the z-stack images utilizing the ImageJ co-localization function. Following subtraction, the remaining extravascular hemoglobin-positive immunofluorescent signal was subjected to threshold processing and quantified using the ImageJ Integrated Density analysis.

For all studies of NNDC and ALS specimens, the same laser settings were utilized (i.e., laser power, amplifier gain and offset, scan speed, optical bandwidth filters, the size of z-stacks), and any variability in background was accounted for utilizing post-image thresholding. To avoid bias, a blinded investigator analyzed all images.

### Statistical analysis

All data were analyzed using Student’s *t* test to analyze differences between ALS and NNDC groups. Correlations were determined using Pearson’s correlation analysis. A *p* value <0.05 was considered statistically significant in all studies. All values expressed as mean ± standard error of the mean (SEM) unless otherwise indicated.

## Results

### BSCB breakdown in human ALS

**Fig. 1:**
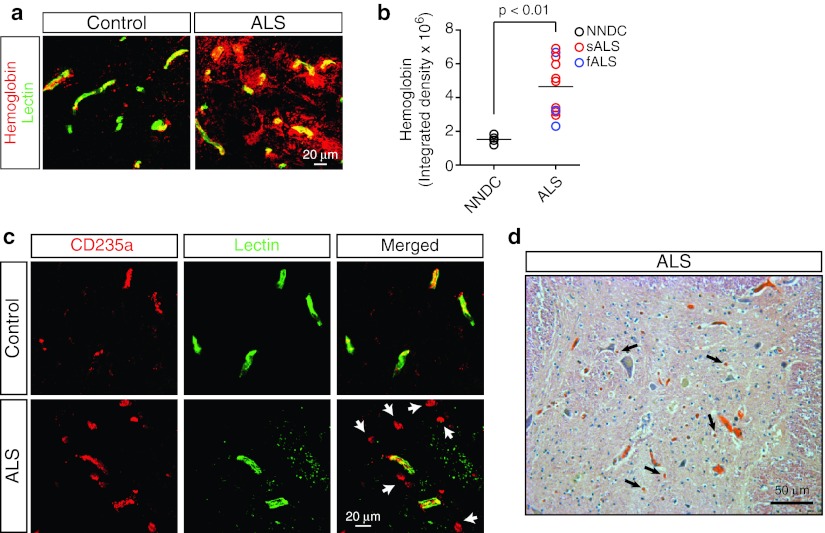
Extravascular deposition of erythrocyte-derived hemoglobin and erythrocyte extravasation in the spinal cord of ALS subjects. **a** Confocal microscopy analysis of erythrocyte-derived hemoglobin (*red*) and lectin-positive capillaries (*green*) in NNDC and sporadic ALS cervical spinal cord anterior horn. **b** Quantification of extravascular hemoglobin deposits in the cervical spinal cord anterior horn. Mean ± SEM, *n* = 5 NNDC, eight sporadic (sALS) and three familial (fALS) cases. **c** Confocal microscopy analysis of CD235a-positive erythrocytes (*red*) and lectin-positive capillaries (*green*) in a NNDC control and sporadic ALS cervical spinal cord anterior horn. **d** Extravasation of erythrocytes in ALS spinal cord anterior horn demonstrated by hematoxylin and eosin staining. In **c** and **d**, *Arrows* denote extravasated erythrocytes in ALS sample

Confocal microscopy analysis of the spinal cervical cord anterior horn gray matter detected multiple extravascular deposits of erythrocyte-derived hemoglobin outside the vascular lumen as indicated by lectin-positive capillary profiles (Fig. 1a). Quantitative analysis revealed a 3.1-fold increase in extravascular hemoglobin deposits in ALS compared to NNDC (controls) showing non-subtracted background levels of intravascular hemoglobin, as indicated by staining with endothelial cell-specific lectin (mean hemoglobin (arbitrary units): ALS, 4,647,513 ± 509,666; *n* = 11 cases; NNDC, 1,525,775 ± 132,292, *n* = 5 cases; for each case six sections and five randomly selected fields per section were analyzed) (Fig. 1b). A similar pattern of extravascular hemoglobin staining was observed in sporadic and familial ALS tissue specimens, but not NNDCs, irrespective of acetone or paraformaldehyde fixation. Simultaneous immunostaining for CD235a, a specific marker for erythrocytes [11, 19, 47] and endothelial cell-specific lectin indicated the presence of sporadic CD235a-positive erythrocytes outside the spinal cord capillaries in ALS specimens, but not controls, as illustrated in Fig. 1c. Extravasation of erythrocytes in ALS has been confirmed by hematoxylin and eosin staining (Fig. 1d), in contrast to their strictly intravascular location in controls (not shown), as expected based on immunofluorescent staining (Fig. 1c).

**Fig. 2:**
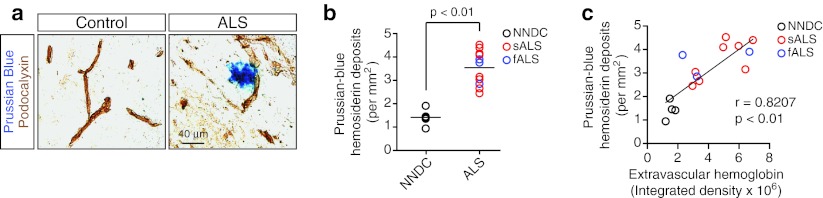
Perivascular hemosiderin deposition in the spinal cord of ALS subjects. **a** Bright field microscopy analysis of Prussian blue-positive hemosiderin deposits (*blue*) and podocalyxin-postive capillaries (*brown*) in NNDC and sporadic ALS cervical spinal cord anterior horn. **b** Quantification of perivascular Prussian blue-positive hemosiderin deposits in the cervical spinal cord anterior horn. Mean ± SEM, *n* = 5 NNDC, eight sporadic (sALS) and three familial (fALS) cases. **c** Positive correlation between perivascular hemosiderin deposits and extravascular hemoglobin deposition in cervical spinal cord anterior horn. Single data points derived from NNDC, sALS and fALS subjects. *r* Pearson’s coefficient

Bright field microscopy analysis of Prussian blue-positive hemosiderin deposits and podocalyxin-positive capillaries revealed perivascular hemosiderin deposits in ALS subjects, but not controls, typically 10–50 μm in diameter predominately surrounding capillaries (≤8 μm in diameter) (Fig. 2a). Quantification of Prussian blue-positive hemosiderin deposits detected approximately a 2.5-fold increase in ALS when compared to NNDCs (mean number of hemosiderin deposits per mm^2^: NNDS, 1.41 ± 0.15, *n* = 5 cases; ALS, 3.54 ± 0.22, *n* = 11 cases; for each case six sections and five randomly selected fields per section were analyzed) (Fig. 2b). In individual subjects, the magnitude of perivascular hemosiderosis positively correlated with the amount of extravascular erythrocyte-derived hemoglobin (*r* = 0.8207, *p* < 0.01) (Fig. 2c), suggesting that lysis of extravasated erythrocytes contributes to the development of perivascular hemosiderin deposits.

**Fig. 3:**
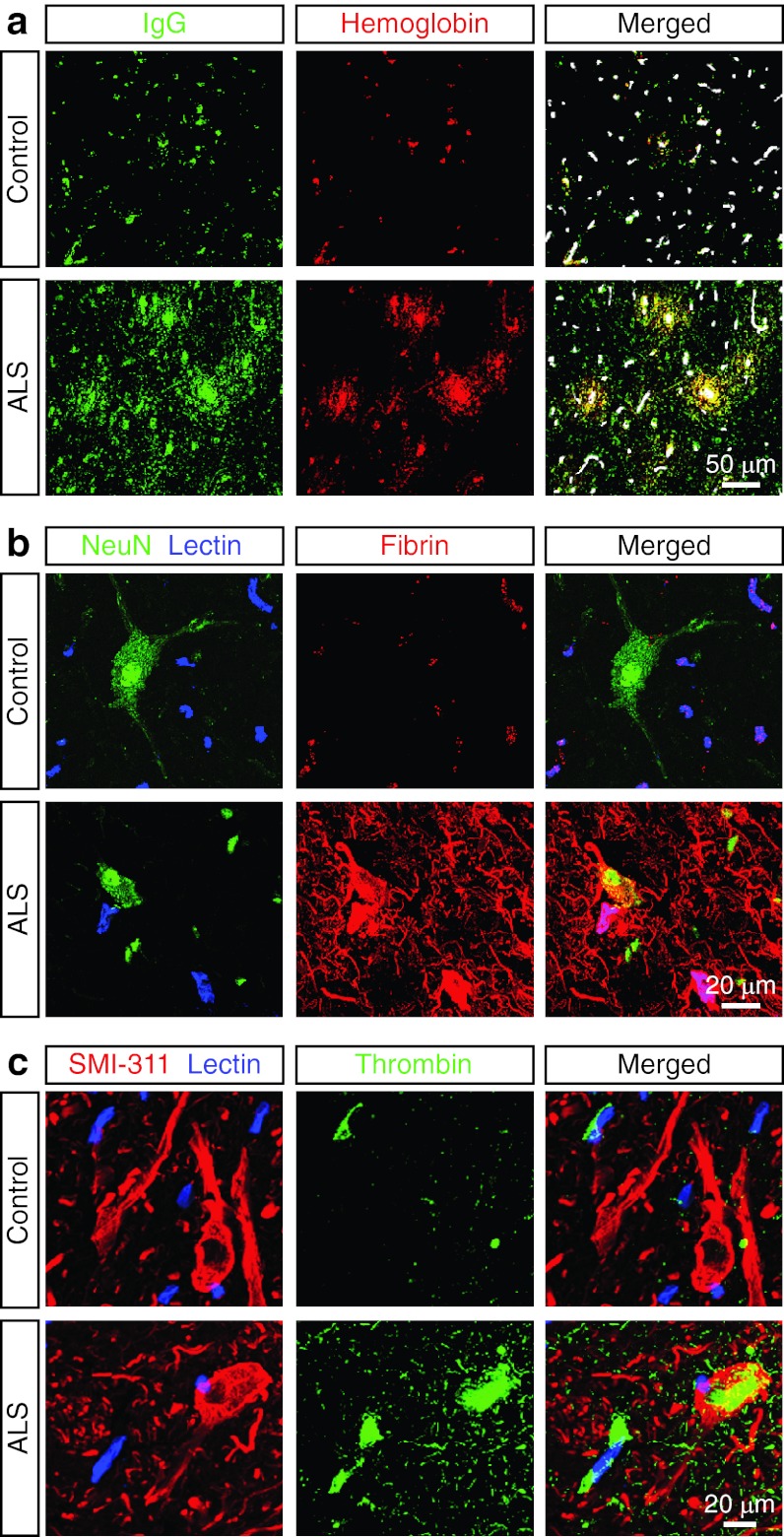
Accumulation of plasma-derived proteins in the spinal cord of ALS subjects. **a** Confocal microscopy analysis of immunoglobulin G (IgG) (*green*) and hemoglobin (*red*) in NNDC and sporadic ALS cervical spinal cord anterior horn. *Merged* extravascular colocalization of IgG and hemoglobin, *white* lectin-positive capillary profiles. **b** Confocal microscopy analysis of plasma-derived fibrin (*red*), NeuN-positive neurons (*green*) and lectin-positive capillaries (*blue*) in NNDC and sporadic ALS cervical spinal cord anterior horn. **c** Confocal microscopy analysis of plasma-derived thrombin (*green*), SMI-311-positive neurons (*green*) and lectin-positive capillaries (*blue*) in NNDC and sporadic ALS cervical spinal cord anterior horn. Representative images are shown from 5 NNDC and 11 ALS cases

Our data show significant perivascular accumulation of immunoglobulin G (IgG) that co-localized with hemoglobin deposits (Fig. 3a), suggestive of BSCB leakage as illustrated by lectin-positive capillaries. Plasma-derived fibrin (Fig. 3d) and thrombin (Fig. 3c) accumulations were also found in motor neuron dense regions in the cervical spinal cord anterior horn gray matter in sporadic ALS subjects, but not controls. Similar accumulates were found in familial ALS subjects. These data further illustrate that BSCB damage results in leakage of blood constituents in human ALS.

### Reduction of spinal cord pericyte population in ALS

**Fig. 4:**
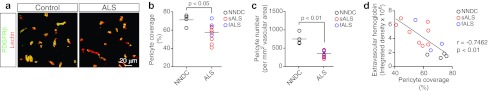
Pericyte capillary reduction in ALS spinal cord correlates with magnitude of vascular disruption. **a** Representative confocal microscopy analysis of PDGFRβ-positive pericytes (*green*) and lectin-positive capillaries (*red*) in NNDC and sporadic ALS cervical spinal cord anterior horn. **b** Quantification of PDGFRβ-positive pericyte capillary coverage in cervical spinal cord anterior horn. Mean ± SEM, *n* = 5 NNDC, eight sporadic (sALS) and three familial (fALS) cases. **c** Quantification of PDGFRβ-positive pericyte cell number per mm^2^ vascular surface area in cervical spinal cord anterior horn. Mean ± SEM, *n* = 5 NNDC, eight sALS and three fALS. **d** Negative correlation between extent of extravascular hemoglobin and pericyte coverage in cervical spinal cord anterior horn. Single data points derived from NNDC, sALS and fALS subjects. *r* Pearson’s coefficient

Using established methods [6, 7, 61, 63], we next determined the percentage of the capillary wall covered by PDGFRβ-positive pericyte cell processes. As reported previously [4, 6, 14, 42, 61, 63], PDGFRβ was not expressed in astrocytes surrounding the vessel wall, as demonstrated by lack of PDGFRβ staining of astrocyte processes positive for glial fibrillar acidic protein, an astrocyte-specific marker. In total, 10 out of 11 subjects with ALS displayed reductions in pericyte coverage. Analysis revealed a statistically significant 19 % reduction in mean PDGFRβ-positive pericyte coverage of cervical anterior horn spinal cord capillaries in ALS subjects when compared to NNDCs (mean pericyte coverage: NNDC, 71 ± 3 %, *n* = 5 cases; ALS, 58 ± 3 %; *n* = 11 cases; for each control and ALS case six sections per case and five randomly selected fields per section were analyzed) (Fig. 4a, b). In addition to reductions in pericyte coverage, 11 out of 11 subjects with ALS displayed reductions in the number of cervical anterior horn gray matter pericytes. Analysis showed approximately a 54 % reduction in PDGFRβ-positive pericyte number in ALS subjects when compared to NNDCs (mean pericyte number: NNDC, 750 ± 78, *n* = 5 cases; ALS, 350 ± 28, *n* = 11 cases; for each control and ALS case six sections per case and five randomly selected fields per section were analyzed) (Fig. 4c). In individual subjects, pericyte coverage correlated negatively with the magnitude of vessel rupture as measured by extravascular hemoglobin abundance (*r* = −0.7462, *p* < 0.01) (Fig. 4d). Collectively, these data suggest that reduced pericyte populations may contribute to microvascular fragility and BSCB breakdown in human ALS subjects.

## Discussion

Our postmortem tissue analysis suggests that BSCB disruption in ALS patients leads to extravasation of erythrocytes in the spinal cord and subsequent accumulation of erythrocyte-derived hemoglobin and iron-containing hemosiderin, as well as extravasation of multiple plasma-derived proteins. We also show that BSCB breakdown in ALS subjects is associated with pericyte loss in motor neuron dense regions of the spinal cord, i.e., the cervical spinal cord anterior horn gray matter. The present study further supports the existence of alterations of the BSCB in ALS subjects. Past studies utilizing both CSF and tissue analyses have suggested possible BBB and/or BSCB disruption in a subset of human ALS subjects varying from 26 to 100 % of cases depending on both the study and the parameter being analyzed (summarized in Table 2).

**Table 2:** Prior studies suggestive of vascular disruption in sporadic and familial amyotrophic lateral sclerosis

Parameter(s)	Key findings	Proportion of subjects with barrier dysfunction (if applicable)	Reference
Cerebrospinal fluid analyses			
Total protein	Elevated CSF total protein	26 % (6/23)	[29]
Total protein	Elevated CSF total protein	*n* = 14	[53]
Plasma-derived protein	Increased albumin, IgG or total protein	46 % (41/90)	[40]
Plasma-derived protein	Increased complement C_3_c in CSF	*n* = 13	[2]
Plasma-derived protein	Elevated CSF/serum albumin ratio	100 % (4/4)	[57]
Plasma-derived protein	Elevated CSF/serum albumin ratio	46 % (17/37)	[3]
Plasma- derived protein	Elevated CSF/serum albumin ratio	50 % (15/30)	[45]
Plasma-derived protein	Elevated CSF/serum albumin ratio	28 % (19/69)	[10]
Postmortem spinal cord analyses			
Plasma protein deposits	Increased spinal cord and motor cortex deposition of plasma-derived IgG and C_3_	Spinal cord: 38 % (6/16) Motor cortex: 38 % (5/13)	[17]
Plasma protein deposits	Increased neuronal uptake of plasma-derived IgG	Spinal cord: 87 % (13/15) Motor cortex: 55 % (6/11)	[20]
Tight junction proteins	Decreased ZO-1 and occludin expression in ALS	*n* = 34	[31]
Matrix metalloproteinase	Increased MMP-9 activity in frontal and occipital cortices and cervical, thoracic and lumbar spinal cord	Cervical: 100 % (9/9) Thoracic and lumbar: 78 % (7/9) Frontal and occipital cortex: 100 % (9/9)	[41]
Inflammatory infiltrate	Increased number of spinal cord T cell lymphocytes	100 % (8/8)	[58]
Inflammatory infiltrate	Increased number of spinal cord T cell lymphocytes	70 % (18/27)	[20]
Inflammatory infiltrate	Increased number of spinal cord dendritic cells and transcripts	100 % (5/5)	[32]
Serum analyses			
Matrix metalloproteinase	Elevated MMP-9 levels in ALS serum samples	MMP-9 Levels: *n* = 14 MMP-9 Activity: 65 % (9/14)	[8]
Matrix metalloproteinase	Elevated pro- and active-MMP-9 in ALS serum samples	*n* = 25	[16]
Matrix metalloproteinase	Elevated levels of MMP-2 and MMP-9 in ALS serum samples	MMP-9 levels: 70 % (21/30)	[49]

At a molecular level, transcriptional analysis has demonstrated that the tight junction proteins of the BSCB, i.e., zonula occludens-1 (ZO-1) and occludin, are reduced in sporadic and familial ALS cases in the lumbar spinal cord suggesting a potential mechanism for barrier disruption [31]. Elevations in matrix metalloproteinase-9 (MMP-9), an enzyme known to chronically degrade endothelial tight junctions [7], have also been detected in ALS serum samples [8, 16, 49] and postmortem brain and spinal cord specimens [41]. MMP-9 has been linked to degradation of the tight junction proteins and extracellular basement membrane matrix proteins at the BSCB of ALS patients by an independent study [46]. Our findings suggest that abnormalities may not be restricted to endothelial cells and that reductions in spinal cord capillary pericytes may also contribute to microvascular disruption in human subjects. Importantly, pericytes have been shown to promote endothelial tight junction protein expression, including ZO-1 and occludin [6], facilitate tight junctional alignment [14], and reduce endothelial vesicular uptake and transcytosis of circulating macromolecules [4]. Pericytes under pathologic conditions have been recently demonstrated to be an important source of secreted MMP-9 leading to degradation of both endothelial tight junctions and the basement membrane resulting in vascular fragility [7]. Whether a similar relationship exists in human ALS remains to be determined and should be addressed.

Unlike other neurodegenerative disorders with chronic CNS microhemorrhage, such as Alzheimer’s disease [13, 27], the spinal cord erythrocyte extravasation described in ALS patients in this study was predominately confined to the capillary level. Following extravasation, erythrocytes lyse and liberate both membranous and cytoplasmic components including free hemoglobin [33, 51, 64]. Following lysis of extravasated erythrocytes, degradation of hemoglobin-derived heme within the CNS gives rise to carbon monoxide, bilirubin and iron [33, 64]. This results in local elevations of both free and protein-bound iron including the insoluble-iron storage protein hemosiderin [33, 64]. Hemosiderin deposits found in the present study were much smaller in size (~10–50 μm) than hemorrhages described at the arterial or arteriolar level and/or in Alzheimer’s disease (>100 μm) [13, 27, 56, 69]. Recent studies have demonstrated that circulating plasma penetrates five times faster than the erythrocyte core [56] and that the plasma protein diameter is typically 3 to 3.5-fold larger than the erythrocyte core in CNS parenchyma following vascular disruption [39]. Similarly, we found that the diameter of distribution of extravasated plasma proteins, such as fibrin, was on average 3 to 4-fold larger than that of hemoglobin. This likely reflects a more centrally localized erythrocyte core surrounded by peripheral plasma protein diffusion in human ALS spinal cord tissue.

Hemoglobin is toxic to motor neurons [54] and neural cells expressing different SOD1 mutants [67] in vitro through iron-dependent oxidative injury. In mouse models of ALS [46, 66, 67] and in pericyte-deficient mice with disrupted PDGFRβ signaling in pericytes [63], BSCB disruption with erythrocyte extravasation and/or motor neuron accumulation of extravasated plasma-proteins such as thrombin and fibrin precedes motor neuron injury. Whether small foci of extravasated erythrocytes can contribute to motor neuron injury in the spinal cord of ALS patients, and whether extravasated proteins (e.g., fibrin, thrombin) localize on the surface or inside motor neurons in human ALS, as found in cortical and/or motor neurons in pericyte-deficient mice [4, 6, 63] or transgenic apolipoprotein E4-expressing mice [7], require future experimental investigation.

Perivascular hemosiderin deposits account for perivascular hypointensities of T2*-weighted MRI analysis utilized clinically for the detection of intracerebral microhemorrhage [27, 28, 60]. The small size of spinal cord hemosiderin deposits as described in the present study may pose a significant barrier to the sensitivity and specificity of conventional T2*-weighted MRI imaging pulse sequences in human subjects [27, 28, 60, 68]. Therefore, MRI studies in ALS patients should be interpreted with caution [68]. For example, one brain MRI study failed to detect microhemorrhages that were <100 μm in diameter in a small number of ALS patients [59], possibly due to limited resolution of imaging [27, 60, 68]. Future studies are needed combining MRI and histopathologic analyses to better optimize pulse sequences for the detection of perivascular hemosiderin deposits in ALS patients, especially in the spinal cord, which has yet to be investigated. Whether ALS patients develop BBB disruption with erythrocyte extravasation, hemosiderin deposition, and/or pericyte reductions in motor cortex and affected brainstem motor nuclei remains to be seen.

Greater than 99 % of circulating iron is bound to hemoglobin [23] and, therefore likely contributes to previously reported elevations in CNS iron in human ALS [35, 36, 38, 50]. In addition, we demonstrate accumulation of plasma-derived proteins including IgG, fibrin and thrombin. At present, it is unclear as to whether the presence of vascular disruption alters the clinical course of ALS cases. A possible limitation of any study of human spinal cord tissues is the post-mortem sampling, with results reflecting an end-stage process. Thus, experimental models are needed to better characterize ALS-associated vascular dysfunction and determine whether exacerbating or mitigating the BSCB breakdown and erythrocyte extravasation in accepted experimental models of ALS contributes to motor neuron injury and disease progression during ALS pathogenesis. Sensitive biomarkers should be investigated that detect ALS pericyte reductions and capillary leakage present in spinal cord tissues. Larger studies and continued development of technologies to detect spinal cord microvascular changes hold considerable promise in deducing whether vascular disruption may represent an important variant of ALS and guide therapeutic development accordingly.
